# Male refractory hypospadias with sexual reversal: a case report

**DOI:** 10.1186/s13256-023-04230-3

**Published:** 2023-11-28

**Authors:** Jianfeng Zhao, Gang Chen, Jun Chen, Le Qian

**Affiliations:** 1 Andrology Department of Traditional Chinese Medicine, Sir Run Run Shaw Hospital Affiliated to Zhejiang University School of Medicine, Hangzhou, 310016 Zhejiang China; 2 The Urology Department, The Second Affiliated Hospital of Zhejiang Chinese Medical University, No. 318 Chaowang Road, Gongshu District, Hangzhou, 310005 Zhejiang China

**Keywords:** Case report, Hypospadias, Disorder of sex development, 46,XX

## Abstract

**Background:**

Hypospadias is one of the most prevalent urogenital malformations in clinic. However, some hypospadias may have a more complex disorder of sex development. Usually, hypospadias in these patients is severe. Among them, the 46,XX male sex reversal syndrome is a rare disorder of sex development, and this may be the main reason for this type of hypospadias being difficult to repair.

**Case presentation:**

We present a Han nationality 19-year-old male with failure of repeated repair of hypospadias. No sperm was found on semen analysis. Lingual mucosal graft was carried out for this patient. It still did not succeed after using lingual mucosal graft repair. Karyotype analysis of this patient confirmed 46,XX karyotype.

**Conclusion:**

Hypospadias with 46,XX male sex reversal syndrome is hard to repair. Chromosome karyotype examination in patients with hypospadias is suggested. Genetic testing is recommended. In the future, further research is needed on the pathogenesis of disease and how to treat and prevent it.

## Background

Hypospadias is one of the most prevalent urogenital malformations in male newborns. It is a congenital deformity of the external genitalia, defined as abnormal positioning of the urethral opening caused by abnormal development of the urethral fold and the ventral foreskin of the penis [1]. This condition occurs during fetal development, when the urethral opening does not form properly. A valid hypothesis is that hypospadias is caused by genetic susceptibility and maternal exposure to endocrine disruptors during the first trimester of pregnancy [2]. Hypospadias can be classified into anterior, midshaft, and posterior on the basis of the location of the urethral opening along the underside of the penis. It is indicated that nearly half of cases are anterior, about 30% are middle, while the rest 20% are posterior [3]. Studies have reported that the normal incidence of hypospadias varies greatly from 5 to 50 cases per 10,000 live births. Although its prevalence varies widely between countries and populations, it shows lower prevalence rates in Asia than in European countries and North America [1, 4].

The cause of hypospadias is not entirely clear. It seems that genetic, endocrine, and environmental factors play an important role. With the development of molecular biology, hypospadias appears to be associated with disruption of gene expression [5, 6]. Isolated anatomical defects are responsible for most hypospadias. However, in a minority patients, hypospadias may have a more complex abnormality of disorder of sex development (DSD). Patients with severe hypospadias are associated with dilemmas about gender, and patients with penile, scrotal, and perineal hypospadias should undergo cytogenetic testing [2]. One study showed that 8.5% of patients with proximal hypospadias have a specific diagnosis of DSD [7].

Among those with DSD, 46,XX male sex reversal syndrome is a rare anomaly characterized by chromosomal and gonadal gender inconsistency. The XX maleness is the most famous example of sex reversal syndrome, with one case of XX maleness occurring among approximately 20,000–30,000 newborn boys [8]. Surgical intervention for hypospadias has evolved with the aim of restoring esthetics and function. However, for the management of hypospadias in DSD, there is still no consensus about the best approach.

## Case presentation

A Han nationality 19-year-old male adolescent was referred to our outpatient clinic owing to refractory hypospadias. He was born with penile curvature and hypospadias. The curvature of the penis was cured after surgery. While he had undergone surgery four times to repair hypospadias, this still failed. The psychosocial history was negative. No familial history of diseases was noted. Full physical examination revealed his skin was relatively white and delicate. His height was 167 cm and his body weight was 53 kg, below normal levels. The male external genitalia appeared normal in terms of penis size with thick black pubic hair at its base. Meanwhile, old scars could be seen on the ventral side of the penis, and fistulas with diameter of approximately 1 and 3 mm, respectively, could be seen on it. Voiding cystourethrogram confirmed this and reveled the distal urethral stricture.

B-ultrasound did not detect female genital organs, and the size of testes ranged from 2.1 × 1.4  × 0.8 cm^3^ to 1.9 × 1.6× 0.9 cm^3^ with normal echoes. No obvious abnormalities were found in liver, gallbladder, pancreas, spleen, kidney, ureter, bladder, or prostate. The endocrinological study revealed high levels of both gonadotropins, with a follicle-stimulating hormone level of 15.6 IU/L (normal range 1.50–12.40 IU/L) and luteinizing hormone level of 13.51 IU/L (normal range 1.70–8.60 IU/L). In addition, a high progesterone level of 0.72 nmol/L (normal less than 0.47 nmol/L) and prolactin level of 804.90 mIU/L (normal range 86.0–324.0 mIU/L) were recorded. Estradiol and testosterone were within the normal range. Semen analyses showed azoospermia. Unexpectedly, the karyotype was 46,XX.

The lingual mucosal graft is normally used in redo hypospadias repair after previous repair failure [9]. Lingual mucosal graft urethroplasty was carried out for this patient. Although the surgery was very successful, the patient ended up with the same two urethral fistulas as before, 20 days after surgery.

## Discussion and conclusions

The 46,XX male sexual reversal was first described as Chapelle Syndrome in 1964 [10]. It is one of the rarest sex chromosomal aberrations seen in clinic. The majority of such patients present an apparently normal male phenotype at birth. Usually they will not diagnosed until genital ambiguities or infertility appear after puberty, while some patients are diagnosed earlier during childhood owing to hypospadias or cryptorchidism. The incidence rate of DSD in individuals with congenital hypospadias and cryptorchidism ranges from 17% to 50% [11].

The sexual phenotype is determined by the Y chromosome-related region Y gene in humans. Sex-determining region of Y (SRY) gene acts as a control for male sex development and triggers the formation of male-specific gonads. However, about 90% of 46,XX males have a portion of the Y chromosome, including the SRY gene. This is a result of recombination between the distal portions of the short arms of the X and Y chromosomes. The remaining part of 46,XX males are negative for SRY gene [12, 13]. This patient had a female karyotype but was phenotypically male. He had the male external genitalia and masculinization. It might be that he was SRY-positive.

Even in SRY-positive 46,XX individuals, the phenotype varies greatly from normal male gonads to abnormal secondary sexual characteristics, small testes, hypospadias, and hermaphrodite. About 20% of those who are 46,XX positive for SRY present abnormal external genitalia at birth, and the typical phenotype is hypospadias [14].

Although the SRY gene is considered to be the main regulatory factor for testicular determination, the phenotypic variability displayed in 46,XX sex reversal cases cannot be explained solely by the presence or absence of the SRY gene.

Besides, a minority of 46,XX male whose are SRY negative could also present with normal male phenotype [15]. A number of other genes such as SRY-related high mobility group-box gene (SOX)9, SOX3, nuclear receptor subfamily 5, group A, member 1 (NR5A1)/steroidogenic factor-1 (SF-1), Desert Hedgehog gene (DHH), nuclear receptor subfamily 0, group B, member 1 (NR0B1), Wilm tumor (WT) 1, wingless type MMTV integration site family, member (Wnt) 4, and Wnt 7a have been implicated in the process of gonadal differentiation. Mutations in any of these genes may lead to the development of DSD. The loss of genes related to male sexual development may lead to an undervirilized male [11, 15, 16].

Usually, 46,XX males have normal testosterone levels during adolescence, followed by a decrease in adulthood that can lead to hypergonadotropic hypogonadism [17]. Some patients have normal genitalia and are diagnosed because of infertility. The patient in this case had hypospadias and small testes. He is young, thus the level of testosterone is normal in him. High levels of follicle-stimulating hormone and luteinizing hormone were observed. Even a 46,XX male may have normal external genitalia and masculinization, and engage in sexual intercourse, but lack spermatogenesis. No sperm was found in this patient. Hypospadia in DSD tends to be more severe than usual, with more proximal urethral fistulas and higher incidence of penile curvature [18, 19]. Moreover, the presence of infection and chronic disease can worsen the prognosis of the condition [20]. DSD may be the main reason why this kind of hypospadias is hard to repair. To date, repeated failed repair of hypospadias in 46,XX has not been reported. Unfortunately, we did not conduct further genetic testing on the patient. In the future, more investigations are needed to prove this. Chromosome karyotype examination is necessary for patients with hypospadias. Further studies will aim to identify the exact pathogenesis of the disease. Besides, surgery should be carried out with considerations such as the cause of the DSD, the function and anatomy of the genitals, fertility, and psychosocial factors.

## Data Availability

The datasets used during the current study are available from the corresponding author on reasonable request.

## Abbreviations

DSD: Disorder of sex development
SRY: Sex-determining region of Y
SOX: SRY-related high mobility group-box gene
NR5A1: Nuclear receptor subfamily 5, group A, member 1
SF-1: Steroidogenic factor-1
DHH: Desert hedgehog gene
NR0B1: Nuclear receptor subfamily 0, group B, member 1
WT: Wilm tumor
Wnt: Wingless type MMTV integration site family, member
